# Characteristics and complexity of chronic pain patients referred to a community-based multidisciplinary chronic pain clinic

**DOI:** 10.1080/24740527.2018.1453751

**Published:** 2018-04-19

**Authors:** C. May, V. Brcic, B. Lau

**Affiliations:** a Faculty of Medicine, University of British Columbia, Vancouver, BC, Canada; b Family Medicine, University of British Columbia, Vancouver, BC, Canada; c Department of Anesthesiology, Pharmacology, & Therapeutics, University of British Columbia, Vancouver, BC, Canada

**Keywords:** chronic pain, cross-sectional, social determinants of health, demographics, practice-based research, patient-reported outcome measures

## Abstract

**Background:**

Community-based care fills an important service gap for patients living with chronic pain. Better understanding of unmet patient needs in the community may inform improved policy and resource allocation.

**Aims:**

The aim of this study was to describe patients presenting to a community-based, multidisciplinary chronic pain clinic in Vancouver, British Columbia.

**Methods:**

This is a retrospective cross-sectional study of 935 unique consecutive patients who completed an intake questionnaire between January 2016 and March 2017. All data were patient reported.

**Results:**

Nine hundred thirty-five patient records were analyzed for descriptive characteristics. The mean age of the population was 49.5 (SD = 14.9) years; 70% were female. Approximately 50% of patients lived below the poverty line in Vancouver; 30% were not working due to disability, 51% had pain for more than 5 years, and 63% reported severe functional impairment.

**Conclusions:**

Substantial unmet need is demonstrated in this patient population accessing a community-based chronic pain clinic. The population described is mainly of working age with significant functional impairment, reflecting a high level of need due to severity and duration of symptoms, poverty, and other characteristics described.

## RÉSUMÉ

**Contexte**: Les soins communautaires comblent une lacune importante pour les patients qui souffrent de douleur chronique. Une meilleure compréhension des besoins des patients qui ne sont pas comblés au sein de la communauté pourrait permettre d’améliorer les politiques et l’allocation des ressources.

**But**: Décrire les patients qui se présentent dans une clinique communautaire multidisciplinaire de douleur chronique à Vancouver, Colombie-Britannique (C.-B.).

**Méthodes**: Étude transversale rétrospective de 935 patients uniques consécutifs qui ont répondu à un questionnaire d’admission entre janvier 2016 et mars 2017. Toutes les données étaient déclarées par les patients.

**Résultats**: Les dossiers de 935 patients ont été analysés afin d’en tirer les caractéristiques descriptives. L’âge moyen de la population était de 49,5 ans (É.-C. 14,9) et 70 % étaient des femmes. Approximativement 50 % des patients vivaient sous le seuil de pauvreté à Vancouver, C.–B.; 30 % ne travaillaient pas en raison d’une incapacité, 51 % avaient de la douleur depuis plus de cinq ans et 63 % ont fait état d’une déficience fonctionnelle grave.

**Conclusions**: Des besoins considérables demeurent non comblés au sein de cette population de patients ayant recours à une Clinique communautaire de douleur chronique. La population décrite est principalement en âge de travailler, ce qui démontre un niveau élevé de besoins en raison de la gravité et de la durée des symptômes, de la pauvreté et d’autres caractéristiques décrites.

## Introduction

One in five Canadians suffers from chronic non-cancer pain, which is referred to here as chronic pain.^1^ Chronic pain leads to economic losses estimated at $37 billion Canadian dollars per year in both direct (health care) and indirect costs (e.g., loss of productivity).^2^ In addition, chronic pain treatment is resource intensive, in particular for patients with high levels of functional impairment. For example, an Australian study demonstrated that in a one-year period, emergency department (ED) visits among chronic pain patients were five times greater than for patients with no chronic pain.^3^ The magnitude of the health and socioeconomic impacts of chronic pain has justified the description of a chronic pain epidemic.^4^

Previous studies in Canada have described the clinical and social characteristics of patients with chronic pain presenting to tertiary pain clinics as “complex,” with multiple comorbidities and moderate to severe biopsychosocial and functional impairments.^5–9^ Contributing to the complexity of presentation and treatment of chronic pain is socioeconomic status (SES). Higher severity of chronic pain is reported by patients with lower SES and in patients experiencing homelessness or with lower household income.^10,11^ Research also indicates that patients of lower SES have greater functional impairment compared to patients of higher SES at the same self-reported level of pain intensity.^12^ Indicators of lower SES, such as applying for disability benefits or lower educational attainment, are also predictors of poorer outcome in multidisciplinary treatment programs.^13,14^ Significant or ongoing socioeconomic stressors—such as trauma, ongoing financial hardship, a history of adverse childhood experiences, or pain or injury in childhood—are correlated with increased complexity and worse outcomes.^15^ The wealth of evidence on the impact of chronic stress and social determinants of health underscores the importance of assessing and describing these characteristics in a population with chronic pain.

There is critical unmet need among chronic pain patients in Canada.^16^ The gold standard of care for patients with chronic pain is multidisciplinary treatment; these programs are commonly provided in the hospital setting.^17^ However, access to chronic pain programs in British Columbia is limited due to prohibitive wait lists,^16,18^ compounded by the small number of publicly funded programs.^19^ In British Columbia, income inequality is among the highest in Canada and growing, making noninsured or alternative pain services requiring out-of-pocket expenses unaffordable to many,^20^ even with private or employer-paid extended benefits.^21^ Hence, there is critical need for the development of sustainable, publicly funded, community-based chronic pain services to better serve patient needs.

In Vancouver, CHANGEpain is a chronic pain clinic attempting to fill this service gap. CHANGEpain is community based, multidisciplinary, and “secondary” level—defined as a short-stay health service provided by medical specialists and requiring referral from a primary care provider. Operating since May 2013 and seeing over 5200 new patients since inception (May 2013–March 2018), the clinic was developed to provide comprehensive treatment services to patients suffering from chronic pain, given the inaccessible alternative of hospital-based programs as described above.

For the treatment of chronic pain in British Columbia, public funding through the Medical Services Plan is only available for physician services; other multidisciplinary non–Medical Services Plan treatment options require private payment or extended care coverage. CHANGEpain thus employs a step-by-step approach that includes publicly funded consultations, interventional procedures, group medical visits, and medication management; services insured through extended benefits such as movement therapies and nutrition consultations; and private-pay, uninsured services such as self-management and education programs, advanced pain procedures, and infusion therapies.

This study is the product of practice-based research integrated into clinic workflow that seeks to describe the characteristics and unmet needs of patients referred to this unique community-based clinic that aims to serve patients with insufficient access to care.

We believe that describing characteristics, including SES,^12,14^ of patients attending this clinic is a first step in identifying unmet needs of patients with chronic pain in the Greater Vancouver area of British Columbia. In addition, describing a community-based population of patients with chronic pain can inform efforts to improve quality of care within the clinic and may inform the development of appropriate financially sustainable pain services to meet the needs of these patients.

The primary objective of this study is therefore to describe the patient population referred to a secondary-level, community-based, multidisciplinary chronic pain clinic based in Vancouver, British Columbia, with a focus on socioeconomic characteristics.

## Methods

### Participants

All patients included in this study were first-time consecutive patients referred by their primary care provider to the clinic who completed an intake questionnaire between January 2016 and March 2017 as part of the standard intake process.

Patients were excluded if they were under an active WorkSafeBC claim or were seen at the clinic solely for an independent medical examination. Intake questionnaires were also excluded from the analysis if they were completed in less than 4 minutes. This cutoff point was determined through an audit of a sample of incomplete questionnaires, which represented duplicates or patients who were not eligible to attend the clinic and also represented patients who only filled out basic contact information and confirmation of treatment eligibility. This early cutoff point does not guarantee that records included were not duplicates; however, we believe that it eliminated a significant portion of duplicates and patients who never attended the clinic. It also allowed all questionnaires with any relevant demographic data to be included in the sample, while eliminating the need for further, technically difficult matching of incomplete intake questionnaires with patient records to determine individual record eligibility for the study.

This study received prior approval by the University of British Columbia’s Clinical Research Ethics Board. Informed consent to use retrospective anonymized data was not required as outlined in TCPS 2 article 3.7A.^22^ Specifically, this study was minimal risk and did not involve intervention but rather secondary use of clinical data, which prohibits obtaining consent retrospectively; finally, the lack of consent is unlikely to have adverse effects on patients and such a study may improve patient care received at the clinic. The study was fully compliant with the consent process outlined by the Tri-Council Policy Statement and the University of British Columbia Clinical Ethics Review Board policy.

### Study design

This is an observational, retrospective, cross-sectional study conducted at a secondary-level, community-based, multidisciplinary chronic pain clinic in Vancouver.

### Data collection

**Figure 1.**
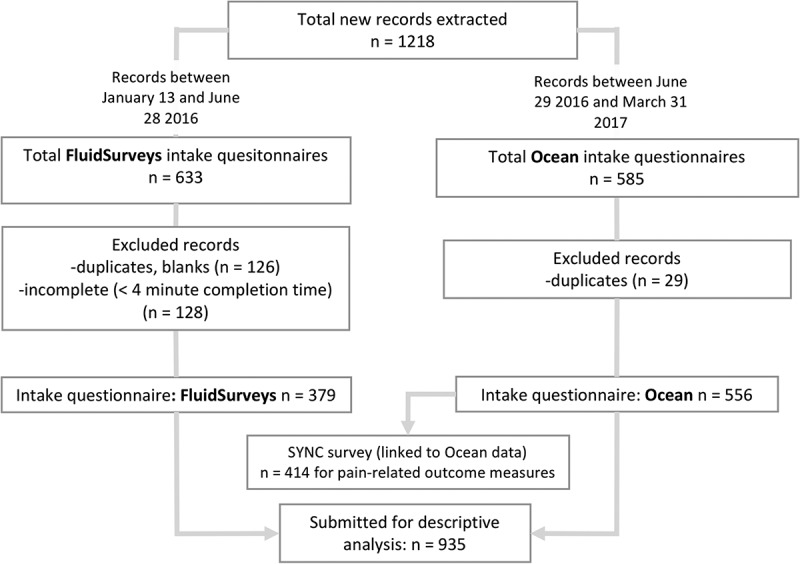
Patient records included and excluded in the study. Also shown are patient records included in FluidSurveys versus Ocean software and the number of EMR-hosted SYNC surveys linked to Ocean intake questionnaire.

All data are self-reported by patients, collected from two surveys administered to patients as part of the routine clinical intake process. Surveys were designed to inform clinical care and contribute to quality improvement and practice-based research. The first of two surveys is an intake questionnaire that is completed by e-mail approximately one month before the patient’s first appointment. This intake questionnaire includes demographic and clinical data including age, gender, marital status, socioeconomic factors, insurance coverage, cause and duration of pain, injury, surgical, medication, and medical history. During the study period, the same intake questionnaire was hosted consecutively on two different online survey platforms: on Fluidsurveys from January 13, 2016, to June 28, 2016 (*n* = 379), and on Ocean from June 29, 2016, to March 31, 2017 (*n* = 556). The upgrade to Ocean was completed due to its ability to securely link intake questionnaire data with the electronic medical record (EMR) (SYNC data) so that patient-reported data could be more readily used in the clinic visit and the practice population data could be more easily analyzed. The second questionnaire (the SYNC survey) includes pain-related baseline outcome measures and is completed by patients on in-clinic computers at the time of their first appointment; data are stored in the clinic EMR system. We were able to extract and analyze these data after switching to the Ocean platform described above (see Figure 1). The SYNC survey consists of five internationally validated, standardized questionnaires assessing patient-reported outcome measures, which were used for baseline measurement of patient biopsychosocial functioning: The Pain Severity Scale, Brief Pain Inventory (BPI), Patient Health Questionnaire–nine items (PHQ-9), Patient Self-Efficacy Questionnaire (PSEQ), and the Patient Related Symptom Severity Catastrophizing (PRSS-CAT) and Adaptive Coping (PRSS-ACT) questionnaires. Subcategories within the validated questionnaires were not available and thus were not analyzed in this study; a composite score was determined to be sufficient to give an overview of the characteristics and needs of the practice population. As the complexity of care for patients with multiple pain areas became of greater concern to clinicians, a question was added to the SYNC questionnaire to identify patients who identified more than one pain area versus only one pain area.

The SYNC survey is re-administered as a follow-up Re-SYNC survey every 3 months until discharge to identify treatment outcomes. Routine collection and study of follow-up data is under development and not included in this study.

### Data analysis

Data for all questionnaire items were extracted to calculate response rates and the distribution of patient demographics and clinical characteristics.

## Results

Out of 1218 new consecutive patient records obtained between January 13, 2016, and March 31, 2017, 935 new patient records were included in this study (Figure 1). Intake questionnaires excluded (total *n* = 283) were those that were blank, duplicates (*n* = 155), or completed in less than 4 minutes (*n* = 128; Figure 1). The median (interquartile range) time between intake questionnaire completion and SYNC survey completion/initial consultation was 14.4 (4.3–36.6) days.

### Sociodemographics

Patient demographics are shown in Table 1. Seventy percent of the patient population were female, and 12 (1.3%) identified as a nonbinary gender. The age range of patients was 17–90 years with a mean (±SD) age of 49.5 (14.9) years. In addition, 27.9% of patients belonged to a visible minority and 5.2% of patients identified as Indigenous.

**Table 1.** Patient demographics.

Variable (total denominator, *N* = 935 or *N* = 556)	%
Patient demographics and characteristics	
Age, mean (SD) (*n* = 906/935)	49.5 (14.9)
BMI, mean (SD) (*n* = 522/556)	26.4 (7.1)
Gender (*n* = 935)	
Female	69.1
Male	29.6
Nonbinary	1.3
Ethnicity/race (*n* = 934/935)	
Caucasian/European descent	71.8
Visible minority	27.9
Indigenous^a^ (*n* = 935)	5.2
Employment (*n* = 935)	
Full- or part-time work	43.7
Unable to work due to disability	29.9
Education (*n* = 935)	
Without high school education	3.6
High school education	13.8
Some college or university	41.8
Postsecondary credentials	39.8
Marital status (*n* = 935)	
Married	55.6
Never married	25.1
Separated (widowed, divorced, other)	19.3

^a^Includes First Nations with or without status, Metis, and Inuk.

BMI = body mass index.

**Figure 2.**
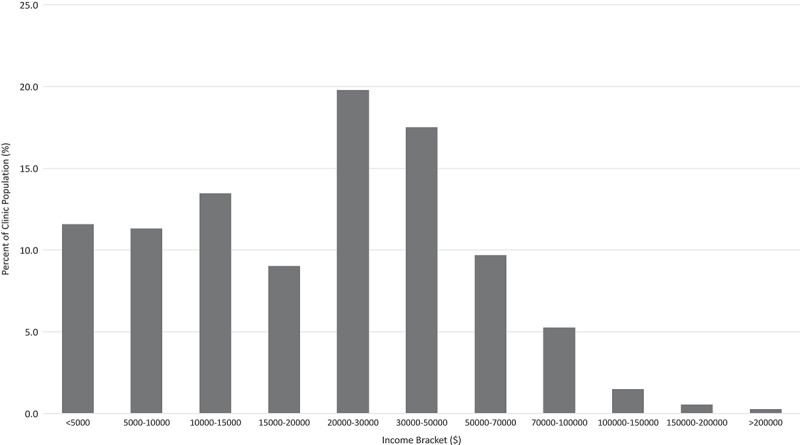
Individual annual income (before taxes) of patients in Canadian dollars (*n* = 747 out of 935 reported income; 188 preferred not to say).

In addition to the demographics reported in Table 1, data demonstrated that poverty and income stress were prevalent. One third of patients (33.7%) were unable to afford “basic bills” at the end of the month. One quarter of patients (25.7%) were on disability income, 8.1% were on welfare, and 21.8% reported ongoing, active litigation. Individual income (before tax; calculated from total household income) ranged from less than $5000 to over $200 000 annually (Figure 2). Of 935 patients, 79.8% of patients reported income. Nearly half (45.2%) of respondents received less than $20 000 annually (approximately representing the living wage in Vancouver), and another 19.8% were in the $20 000 to $30 000 income bracket. Two thirds of all patients (66.8%) reported having extended health insurance, but only 57% of patients who reported earning less than $20 000 annually had extended health insurance, compared to 84% of patients who earned more than $20 000 annually.

The majority of patients did not report significant social isolation; 92% reported at least one person they could confide in. However, 54% of patients reported that they rarely or never had someone who could take them to the doctor when needed.

### Clinical and pain characteristics

Of 935 patients who reported duration of pain, half (50.7%) reported pain for more than 5 years before starting treatment at the clinic (Table 2). At the time of referral, 73.6% of patients were taking at least one prescription medication for their pain, with opioid medication and antidepressants comprising the majority of prescriptions (Table 3). Ninety-one percent of patients reported previously using physiotherapy for their pain before consultation, and 65% used either emergency departments (44%) or walk-in clinics (52%) for pain management (Table 4).

**Table 2.** Pain-related characteristics.

Variables (total denominator, *N* = 935 or *N* = 414)	%
Cause(s) of pain (“select all the apply”) (*n* = 935)	
Car accident	35.7
Other physical strain or injury	17.6
Medical illness	17.0
Repetitive strain	16.7
Sports or recreation injury	14.5
Surgery or medical procedure	13.2
Workplace injury	10.7
I don’t know	28.7
Duration of pain (*n* = 935)	
<6 months	1.8
6 months to <1 year	4.7
1–5 years	42.9
5–10 years	22.7
10–20 years	17.9
>20 years	10.1
Pain during childhood	11.9
Pain distribution (*n* = 315/414)	
More than one pain area	51.1
Only one pain area	48.9
Primary pain areas (*n* = 337/414)	
Head and jaw	14.7
Neck	14.1
Upper back	10.9
Upper arm^a^	10.5
Torso (chest, ribs, abdomen)	5.1
Lower back	25.2
Buttock, pelvis, hip	20.8
Lower leg^b^	10.2
Pain-related stressors (*n* = 855/935)	
Family and relationships	45.8
Work, school, or functional stress/loss	44.7
Financial and legal manners	41.6
Chronic pain	33.9
Self-reported PTSD symptoms/diagnosis (*n* = 935)	26.6

^a^Includes shoulder, upper arm, elbow, forearm, wrist, and hand.

^b^Includes upper leg, knee, lower leg, ankle, and foot.

PTSD = posttraumatic stress disorder.

**Table 3.** Patient-reported current medications at intake.

Medication class (*N* = 926 out of 935)	%
Opioids	
All	33.5
T3, T1, codeine, hydrocodone	10.2
Hydromorphone, morphine	7.2
Fentanyl, oxycodone/contin/neo, percocet	7.2
Tramacet, tramadol	6.8
Buprenorphine, methadone	1.0
Other	0.2
Nonopioids	
Antidepressants	32.9
NSAIDs	29.2
No current pain-related medications	26.6
Anticonvulsants	25.4
Muscle relaxants	17.0
Medications for sleep	16.6
No current medications	6.4
Synthetic marijuana (e.g., nabilone, Sativex)	4.5

NSAIDs = nonsteroidal anti-inflammatory drugs.

**Table 4.** Patient-reported past health service utilization.

Variable (“select all that apply”; *N* = 935)	%
Health care service utilization (*n* = 935)	
Physiotherapy	91
Massage	82
Chiropractic	69
ED or walk-in clinic	65
Self-management strategies (*n* = 545)	59
Walk-in clinic	52
Psychologist/counselor	52
Emergency department	44
Pain management by a GP	44
Naturopathy	35
Pain specialist	34
Kinesiology	29
Nutritionist	27
Occupational therapy	23
Private-pay therapies	20
Osteopathy	15
Indigenous healer	7

ED = emergency department; GP = general practitioner.

Past medical, surgical, and injury data revealed that the majority of patients (60.8%) reported a past or current medical condition (most common were hypertension and inflammatory/irritable bowel disease/syndrome), previous surgery (78.8%; most common was abdominal surgery), and previous injury (88.6%; most common was a car accident or fall; Table 5).

**Table 5.** Patient-reported past medical, surgical, and injury history.

Variable (“select all that apply”; *N* = 935)	%
Past medical history (*n* = 926)	
Hypertension	14.2
Irritable bowel disease/syndrome	13.6
Thyroid disease	13.3
Lung disease	11.9
Anemia	10.3
Osteoporosis	9.8
Cancer	7.5
Diabetes	7.0
Cardiovascular disease	5.5
Kidney disease	2.5
Stroke	1.6
No medical condition	40.2
Surgical history (*n* = 913)	
Other surgery	42.6
Abdominal	25.2
Knee	11.3
Hand or wrist	10.5
Foot or ankle	8.6
Lumbar	7.6
Pelvic	7.0
Shoulder	4.7
Hip	3.7
Chest or thoracic	3.7
Arm or elbow	3.4
Leg	3.3
Cervical	3.1
Other spinal	1.5
No surgery	21.2
Injury history (*n* = 913)	
Car accident	54.0
Fall	38.6
Sports injury	34.8
Fracture	32.8
Repetitive strain	28.3
Other injury	20.4
Work injury	19.6
No injury	11.4

**Table 6.** Baseline pain-related outcome measures.

Variable (*N* = 414)	% or mean (SD)
BPI (*n* = 371)	
Mean (SD)	44.16 (14.44)
0–13 (mild pain interference)	4
14–41 (moderate pain interference)	33
42–70 (severe pain interference)	63
Pain Severity Scale (*n* = 381)	
Mean (SD)	5.44 (1.93)
0 to 3 (mild intensity)	15.7
4 to 6 (moderate intensity	50.7
7 to 10 (severe intensity)	33.6
PHQ-9 (*n* = 374)	
Mean (SD)	12.17 (6.59)
0 to 9 (none–mild depression)	40.1
10 to 19 (moderate depression)	44.7
20 to 27 (severe depression)	15.2
PRSS-ACT, mean (SD) (*n* = 337)	2.80 (0.93)
PRSS-CAT, mean (SD) (*n* = 344)	2.61 (1.15)
PSEQ, mean (SD) (*n* = 356)	29.2 (13.56)

BPI = Brief Pain Inventory; PHQ-9 = Patient Health Questionnaire–nine items; PRSS-ACT = Pain-Related Self-Statement Scale (Active Coping); PRSS-CAT = Pain-Related Self-Statement Scale (Catastrophizing); PSEQ = Patient Self-Efficacy Questionnaire.

Of the 414 EMR SYNC surveys linked to Ocean intake questionnaires, Pain Severity Scale (*n* = 381/414) data indicated that the majority (84.3%) of patients had moderate to severe pain intensity (4–10/10).^23^ PHQ-9 (*n* = 374/414) data indicated that three in five patients (59.9%) reported symptoms indicative of moderate to severe clinical depression (10–27/27).^24^ BPI (*n* = 371/414) results demonstrated that two thirds (63%) of patients experienced severe functional disability (with a score greater than 42/70;Table 6).^25^ Mean (SD) PSEQ (/60) was 29.2 (13.56), greater than a score of 25/60 seen in a sample of 4645 chronic pain patients.^26^ Mean (SD) PRSS-ACT (/5) was 2.80 (0.93) greater than a score of 2.7/5 seen in a sample of 3713 chronic pain patients.^26^ Mean (SD) PRSS-CAT (/5) was 2.61 (1.15), less than a score of 2.7/5 seen in a sample of 4051 chronic pain patients (Table 6).^26^ Fifty-one percent of respondents also reported pain in more than one area of the body.

## Discussion

This study demonstrates the feasibility of recording and analyzing multiple key characteristics of patients with chronic pain attending a secondary-level, multidisciplinary, community-based chronic pain clinic.

In Vancouver, the Market Basket Measure for a single person of $19 536 defines after-tax income required to afford shelter, food, clothing and footwear, transportation, and other household needs and is a cutoff used to define the poverty line.^20^ At least 45.4% of our patients made less than this amount per year after taxes and thus are considered below the poverty line.^20^ Furthermore, 11.6% of our patients earned less than $5000 annually, which is more than the 8.1% of British Columbians in this income cohort.^27^ This is a concerning finding, because low income has been shown to be associated with more refractory forms of pain such as chronic widespread pain.^28^ Furthermore, disability from chronic pain may limit employment, further reducing much-needed income for these patients.^29^ However, in a cross-sectional study, it is impossible to presume a definite direction of association between poverty and pain. Finally, 188 of 931 patients did not report income, which may be due to patients not knowing household income, stigma associated with poverty leading to income nondisclosure, or patients with high income being protective of disclosure.

Patients referred to multidisciplinary pain clinics carry a significant burden of disability and psychosocial consequences due to pain,^8,30^ which was demonstrated in this population. Thirty percent were unable to work due to disability, and almost two thirds (63%) reported severe functional impairment due to pain (defined as BPI > 42/70). Additionally, 54% of patients reported not having a person to help them get to a doctor regularly, which may indicate further barriers in accessing care.

Experiences of chronic pain often include increased emotional distress and impaired emotional processing that may also influence the outcome of pain therapies.^31^ Given that the majority (60%) of our patients met criteria for moderate to severe depression, there is great need to provide targeted and timely therapies for both depression and pain.^32^

Patients presenting to this secondary clinic reported high levels of pain severity, with 84.3% of patients suffering moderate to severe chronic pain, 50.7% of patients having pain of five or more years in duration at the time of consultation, and 11.9% reporting pain since childhood. Most patients already tried alternative care options at the time of referral; 96% tried one or more physical therapy modalities (kinesiology, physiotherapy, massage, etc.). The majority (65%) also reported reliance on episodic urgent care including the ED and walk-in clinics. At the first consultation, 73.4% of patients were managing pain with medications and 33.5% were using opioids.

CHANGEpain aims to reduce reliance on medications by providing nonpharmacological, multidisciplinary treatment alternatives. A priority action in the context of the opioid crisis is developing solutions that reduce reliance on opioids to treat pain.^33^ We believe that the high level of medication reliance, high levels of disability affecting a working-age population, and financial barriers identified by this patient population are a further call for appropriately funded and timely multidimensional solutions. Furthermore, services that treat chronic pain problems earlier and more effectively^34,35^ may prevent long-term biopsychosocial complications.^9,35^

In summary, this clinic was developed to address neglected needs of patients in the community suffering from chronic pain. Initially, both demographic data and pain-related outcomes were collected to inform clinical practice. Software and database capabilities at the clinic allowed us to study trends in patient characteristics and outcomes, and a small practice-based research team was established to analyze data and improve clinic procedures to meet both clinical and research needs. Challenges faced in this study outlined below (such as the limitations with initial FluidSurveys software) led to subsequent improvements that led to improved data quality without affecting clinical workflow. We hope that this process example encourages other clinicians to develop, study, and share practice-based data to contribute to a growing knowledge base of community-based pain services.^7,36^ Limitations listed below reflect challenges in our resource-limited setting, with improvements and mitigating factors described.

## Limitations

A primary limitation in this study was the inability to link SYNC survey data (stored in the EMR) to intake questionnaire data until midway into the data period when codes were written with the new survey platform, Ocean, to reliably and securely integrate these surveys. Due to this limitation, our sample size for pain-related, patient-reported outcome measures (*n* = 414) was lower than the sample for sociodemographic data collected in the intake questionnaire (*n* = 935), because unlinked SYNC survey data could not be extracted for the analysis. The exclusion of 283 records (Figure 1) also contributed to a reduced sample size; this was due to patients starting, stopping, and then restarting the survey online, which created an extra blank or partially complete copy, inflating the number of excluded charts. We were nevertheless able to demonstrate the feasibility of practice-based research, and with ongoing data collection and improvements based on these findings, further research will be possible.

As a cross-sectional study, no causality may be inferred. Some patients did not complete one or both questionnaires, which may indicate a selection bias toward patients who were literate, fluent in English, and able to spend the necessary time to complete the questionnaires. However, this was mitigated by clinic volunteers who were intermittently available to help patients complete the questionnaires in person if incomplete or if they were unable to complete it at home. For incomplete intake questionnaires hosted on Fluidsurveys (*n* = 128 were excluded with <4 minutes completion time), it is unlikely that excluded surveys were representative of the practice population, because a sample audit of incomplete Fluidsurveys questionnaires identified that excluded surveys were from patients who met our exclusion criteria or those unable to attend a first consultation. Analysis of intake data through Ocean software confirmed that all patients who attended a first appointment had a completed intake questionnaire, likely due to the success of software reminders and clinic procedures that supported patients to complete the Ocean intake questionnaire at home or in clinic if unable to do so. However, for unknown reasons (reported by software as a “random error”), only 414 SYNC charts were extractable through Ocean. This may be due to software error or missing or corrupted files.

Although baseline outcome measures describing pain states were completed on the day of the first consultation, patients completed the intake questionnaire (comprised of sociodemographic data) a median (interquartile range) of 14.4 (4.3–36.6) days prior to the first appointment. This was important for clinic workflow to ensure that the data were available to the clinician by the time of first appointment. Some patient characteristics may have changed within this time interval, however, given that half of our patient population had pain for 5 years or more and that the content of the early survey was on less time-sensitive content, we believe that the data appropriately represent patients’ conditions and circumstances at the time of intake, prior to treatment at the clinic.

Some questions were more prone to being answered incorrectly due to use of unknown or technical language (e.g., “previous treatment with radiofrequency lesioning”) or ambiguity (e.g., cause of pain due to “traumatic event”). However, the likelihood of misclassification error was primarily a concern with more complex questions pertaining to past medical and surgical history that we excluded from our analysis; thus, we do not believe that this has a substantial impact on our findings. Items with poor response rates or lack of relevance were excluded from the study; this included, for example, self-reported susceptibility to infection, pregnancy, Internet access, needle phobia, and patient goals.

Although patient self-reported data are increasingly recognized as valuable in practice-based research,^37^ any self-reported data are vulnerable to recall bias. This is particularly relevant in self-reported past experiences and medications; thus, we do not draw substantial conclusions from these results and will use the data to inform further research and more robust future data collection. Notably, a previous study found that patients with chronic widespread pain recall much of their past experiences as attributed to their pain experience, especially childhood experiences.^38^ Secondly, it has been shown that patient recall of medications usually underreports what is listed in the pharmacy database^39^; we recommend linkage with Pharmanet data for future research.

## Conclusion

This secondary-level, community based, multidisciplinary pain clinic fills an important service gap in the community for patients with complex chronic pain who have tried other options, including tertiary pain clinics, yet present with persistent unmet medical need due to ongoing severe pain and disability. Financial stress is prevalent in our practice population; thus, appropriate interventions for chronic pain should also address socioeconomic barriers to facilitate equitable access to gold standard, evidence-informed, multidimensional pain care. Future research should focus on outcomes of pain therapies to meet the unique needs of different groups of patients with chronic pain.
